# Analysis of coupling between magnetic dipoles enhanced by metasurfaces for wireless power transfer efficiency improvement

**DOI:** 10.1038/s41598-018-33174-8

**Published:** 2018-10-05

**Authors:** Hemn Younesiraad, Mohammad Bemani

**Affiliations:** University of Tabriz, Department of Electrical and Computer Engineering, Tabriz, 5166616471 Iran

## Abstract

In this paper, we investigate the possibility of improving efficiency in non-radiative wireless power transfer (WPT) using metasurfaces embedded between two current varying coils and present a complete theoretical analysis of this system. We use a point-dipole approximation to calculate the fields of the coils. Based on this method, we obtain closed-form and analytical expressions which would provide basic insights into the possibility of efficiency improvement with metasurface. In our analysis, we use the equivalent two sided surface impedance model to analyze the metasurface and to show for which equivalent surface impedance the WPT efficiency will be maximized at the design frequency. Then, to validate our theory, we perform a full-wave simulation for analyzing a practical WPT system, including two circular loop antennas at 13.56 MHz. We then design a metasurface composed of single-sided CLSRRs to achieve a magnetic lensing based on the calculated equivalent surface impedance. The analytical results and full-wave simulations indicated non-radiative WPT efficiency improvement due to amplifying the near evanescent field which can be achieved through inserting the proposed metasurface.

## Introduction

Electricity supply is one of the most important challenges with the increasing expansion of electrical appliances and changes in the human lifestyle. Wireless Power Transfer (WPT) is one of the most useful and practical solutions for charging a variety of electronic devices^1^. The earliest suggestions of WPT were proposed by Tesla in 1891, but his prototype was not safe and practical due to the lack of standard radio frequency technology at that time^2^. Wireless power transfer can be used to charge personal electrical devices (smartphones, tablets, laptops), electric vehicles^3^, medical implanted devices^4,5^ and so on. In 1963, the first microwave WPT was demonstrated by Brown^6^. Wireless power systems, mainly fall into two categories: far-field transmission and near-field transmission. In the far-field region or radiative transmission, also called power beaming, power is transferred by beams of electromagnetic radiation, such as microwaves or laser beams. In this type of WPT, the energy is transferred through absorption and scattering in the atmosphere. which requires a direct line of sight between the source and the device^6–8^. The main challenge in this WPT scheme is human electromagnetic exposure safety and WPT efficiency issues. Radiative WPT is suitable for space or military applications. In the near-filed region or non-radiative WPT, energy is transferred through near-field electromagnetic waves from the source to receiver^9^. In this WPT scheme, the operational distance between the transmitter and receiver is far shorter than the wavelength which is suitable for charging electrical devices with short and midrange distance. In this region, the oscillating electric and magnetic fields are decoupled and power can be transferred via electric near-fields via capacitive coupling (electrostatic induction)^10^ between metal electrodes, or via magnetic near-fields by inductive coupling (electromagnetic induction) between coils of the wire^11^. The other type of WPT schemes is magnetic resonant coupling power transfer (MRPT), where the energy can be transferred between two magnetically coupled resonant devices with the same resonance frequency.

In^12^, using self-resonance coils in a strongly coupled regime the efficiency of non-radiative WPT over a distance of up to 8 times the radius of the coils has been demonstrated experimentally with about 40% efficiency over a distance of 2 meters. Since in the near field of magnetic dipole antennas the E/H ratio can be strongly suppressed, thus the interaction with biological and other environmental objects declines, which can ensure human electromagnetic exposure safety^13^. Influential factors in WPT are the Q-factor of the transmitter and receiver resonators as well as the other coupling efficient between these resonators^1^. High Q-factor resonator design can be partly enhanced the WPT efficiency^14–17^. WPT efficiency decreases by 1/*d*^6^, with d being the distance between the transmitter and receiver coils. Since non-radiative WPT basis is based on energy transmission through near-field coupling between coils, so WPT efficiency can be enhanced by manipulating electromagnetic waves. One of the suitable solutions to WPT efficiency improvement is using the multiple passive elements cooperatively for example to form near-filed guiding structure^18^. Another solution to manipulating electromagnetic waves is using metamaterial which possesses the property of evanescent wave amplification^19,20^. Metamaterials are artificial periodic structures arranged in a regular array across a region of space having extraordinary properties not normally found naturally, including negative refractive index, near zero index, etc.^19,20^. The artificially structured metamaterials with negative refractive index are called perfect lens first introduced by Veselago in 1968^21^ and further developed by Pendry *et al*. in 2000^22^. In WPT systems, use of the perfect lens idea is a good starting point for improving the coupling between two coils via refocusing the flux into the receiver. Recently, many studies have been conducted theoretically^13,23,24^ and experimentally^25–31^ on the effect of a variety of metamaterials and metamedia on WPT performance. For the first time, Wang experimentally implemented a WPT system using a mu-negative (MNG) metamaterial. In^13^, the effect of metamaterial on WPT efficiency improvement has been investigated employing a simplified geometry such as an infinitely large slab and point dipoles for the source and receiver to obtain a closed form and analytical expressions. Another original approach to manipulating electromagnetic wave for WPT efficiency enhancement is use of metasurface instead of metamaterial slab^18,32,33^. Three-dimensional metamaterials can be extended by engineering sub-wavelength resonators, whose spectral response can be controlled independently by tailoring the element size and shape into a two-dimensional pattern at a surface or interface. This surface version of a metamaterial has been given the name of metasurface^19,20^. For many applications, metasurfaces can be used in place of metamaterials. Metasurfaces have the advantage of taking up less physical space than do full three-dimensional metamaterial structures; consequently, metasurfaces offer the possibility of less-lossy structures. On other hand, scattering by such a metasurface is best characterized by generalized sheet transition conditions (GSTCs), in contrast to the effective medium description used for a metamaterial. The generalized sheet-transition conditions can also be cast in the form of impedance-type boundary conditions that has been used in this work. The generalized sheet-transition conditions along with Maxwell’s equations are all that are required to analyze the interaction of the fields with the metasurface when the fine structure of the spatial field variation is not required. Indeed, the nature of metamaterials is the exact design of constitutive parameters, whereas metasurfaces can be treated as a modification of boundary condition since they possess theoretically vanishing thickness^19^.

In this paper, we propose a WPT system based on near-field electromagnetic waves manipulation using a metasurafce embedded between the transmitter and receiver coils. We laso present a complete theoretical analysis of this system. We use a point-dipole approximation to calculate the fields of the coils. Based on this method, we obtain closed-form and analytical expressions which would provide initial insights into the possibility of efficiency improvement with metasurface.

## Results

### Calculating mutual inductance in the point-dipole approximation

**Figure 1:**
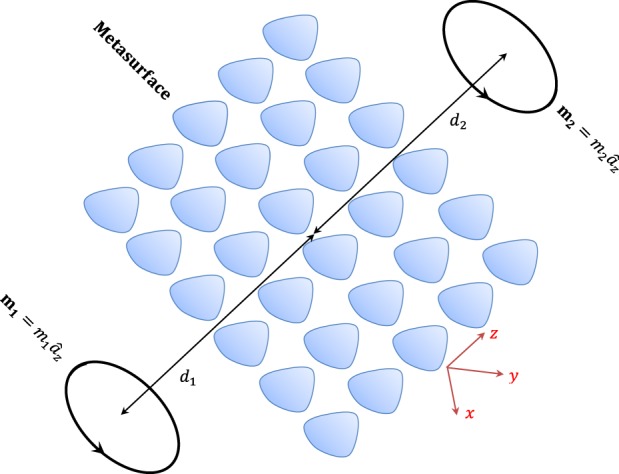
WPT system including transmitter and receiver coils (approximated as magnetic dipoles) separated by distance *d* = *d*_1_ + *d*_2_ through a metasurface.

**Figure 2:**
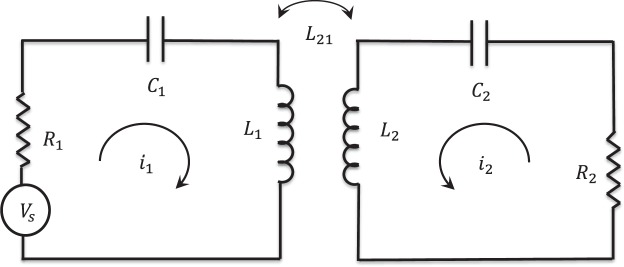
Equivalent Circuit model of magnetic resonant coupling based wireless power transfer system.

To investigate the metasurface effect on the wireless power transfer performance, we should calculate the mutual inductance *L*_21_ in response to the presence of a metasurface between two current-carrying coils. Both coils are negligibly small in comparison with the spatial scale of the magnetic gradient. Figure 1 demonstrates the wireless power transfer system, including two single-turn coils through a metasurface. The metasurface is perpendicular to the z axis. The coils are positioned on the z axis, with their magnetic dipole moments oriented in the z-direction. The transmitter and receiver coils are positioned within the distance *d*_1_ and *d*_2_ from the metasurface, respectively. The magnetic moment of the wire coils corresponds to the current i and area of the coils as $m=i\pi {a}^{2}\widehat{n}$, where $\widehat{n}$ and a are the normal vector perpendicular to the surface of the coils and radius of the coil, respectively. The coils revealed in Fig. 1 can be approximated as magnetic dipoles assuming a significantly small radius for the coils and the diameter of the constituent wire of the coils. In Fig. 2 an equivalent simplified circuit model is provided for Fig. 1, where *R*_1_ and *R*_2_ denote the equivalent resistance (including ohmic and radiation). Conduction loss resistance *R*_*cond*_ is given by the well-known ohm’s law ${R}_{cond}=\frac{l}{{\rm{\sigma }}A}$ where *σ* is the electrical conductivity of the conductor and A is the conductor cross-sectional area. The radius of each loop is assumed to be much smaller than both the free-space wavelength and the spatial scale of variation of the magnetic fields generated by all other loops. Since the source magnetic dipole is perpendicular to the z axis. so it will generate only *TE* waves. Because of the *TE* nature of its field, a vertical magnetic dipole (VMD) can be characterized by *H*_*z*_ only^34^. A metasurface can be represented by an equivalent homogenized two dimensional surface with the electric conductivity tensor ${\overline{\overline{\sigma }}}_{e}$ and magnetic conductivity tensor ${\overline{\overline{\sigma }}}_{m}$. In this work, we used the two-sided surface impedance model to analyze the metasurface. If the period a of an electric MS is small compared to the wavelength, the tangential component of the electric field in the grid plane averaged over the unit cell area is simply proportional to the averaged electric current induced in the grid. This averaged current is equal to the jump of the tangential component of the surface averaged magnetic field across the MS plane, while the averaged tangential electric field experiences no jump. Accordingly, the boundary conditions at the metasurface are^19,20^

1 $${\widehat{a}}_{z}\times {H}_{{0}^{-}}^{{0}^{+}}=\frac{1}{2}{\overline{\overline{{\rm{\sigma }}}}}_{e}\cdot ({E}_{T}^{{0}^{+}}+{E}_{T}^{{0}^{-}}).$$

2 $${\widehat{a}}_{z}\times {E}_{{0}^{-}}^{{0}^{+}}=0.$$

where, ${\overline{\overline{\sigma }}}_{e}$ = ${\overline{\overline{Y}}}_{s}$ and ${\overline{\overline{Y}}}_{s}$ is the grid admittance tensor. In (1), the subscript ‘T’ represents the transverse component of the electric field. The axial component of the electric and magnetic field using Chew’s approach^34^ can be written as:

3 $${H}_{z}=\frac{-j{m}_{1}}{8\pi }{\int }_{-\infty }^{\infty }\,d{k}_{\rho }\frac{{k}_{\rho }^{3}}{{k}_{z}}{H}_{0}^{\mathrm{(1)}}({k}_{\rho }\rho )({e}^{j{k}_{z}z}+{R}_{TE}{e}^{-j{k}_{z}z}){e}^{j{k}_{z}{d}_{1}}.$$

in the −*d*_1_ < *z* < 0 and

4 $${H}_{z}=\frac{-j{m}_{1}}{8\pi }{\int }_{-\infty }^{\infty }\,d{k}_{\rho }\frac{{k}_{\rho }^{3}}{{k}_{z}}{H}_{0}^{\mathrm{(1)}}({k}_{\rho }\rho )({T}_{TE}{e}^{j{k}_{z}z}){e}^{j{k}_{z}{d}_{1}}.$$

in the *z* > 0, where ${k}_{z}=\sqrt{({k}_{0}^{2}-{k}_{\rho }^{2})}$ and *R*_*TE*_ and *T*_*TE*_ are reflection and transmission coefficients, respectively, while ${H}_{0}^{\mathrm{(1)}}({k}_{\rho }\rho )$ is Hankel function. To simplify the calculations, we used the spectral components of all fields according to^34^ to reduce the problem to one dimension of plane waves in the z-direction through the metasurface.

5 $$\overline{H}(\rho ,\phi ,z)={\int }_{-\infty }^{\infty }\,d{k}_{\rho }\tilde{H}({k}_{\rho },z,\phi )$$

6 $$\overline{E}(\rho ,\phi ,z)={\int }_{-\infty }^{\infty }\,d{k}_{\rho }\tilde{E}({k}_{\rho },z,\phi )$$

The other required components of the electric and magnetic fields are expressed through the following formulas of Chew^34^:

7 $${\tilde{E}}_{y}=-\,\frac{j\omega \mu }{{k}_{\rho }^{2}}\frac{\partial {\tilde{H}}_{z}}{\partial x}$$

8 $${\tilde{H}}_{x}=\frac{1}{{k}_{\rho }^{2}}\frac{{\partial }^{2}{\tilde{H}}_{z}}{\partial x\partial z}$$

The spectral components of the fields given by (3) and (4) can be written as:

9 $${\tilde{H}}_{z}=\frac{-j{m}_{1}}{8\pi }\frac{{k}_{\rho }^{3}}{{k}_{z}}{H}_{0}^{\mathrm{(1)}}({k}_{\rho }\rho )({e}^{j{k}_{z}z}+{R}_{TE}{e}^{-j{k}_{z}z}){e}^{j{k}_{z}{d}_{1}}.$$

in the −*d*_1_ < *z* < 0 and

10 $${\tilde{H}}_{z}=\frac{-j{m}_{1}}{8\pi }\frac{{k}_{\rho }^{3}}{{k}_{z}}{H}_{0}^{\mathrm{(1)}}({k}_{\rho }\rho )({T}_{TE}{e}^{j{k}_{z}z}){e}^{j{k}_{z}{d}_{1}}.$$

in the *z* > 0.

For determining the unknown reflection and transmission coefficients, substituting Eqs (9 and 10) in (1 and 2) and using (5–7), we obtain:

11 $$-{k}_{z}({T}_{TE}-\mathrm{(1}-{R}_{TE}))=\frac{\omega \mu }{2{Z}_{s}}({T}_{TE}+\mathrm{(1}+{R}_{TE})).$$

12 $${T}_{TE}=\mathrm{(1}+{R}_{TE}\mathrm{).}$$

The coefficients of interest are:

13 $${R}_{TE}=\frac{-\omega \mu }{2{k}_{z}{Z}_{s}+\omega \mu }.$$

14 $${T}_{TE}=\frac{2{k}_{z}{Z}_{s}}{2{k}_{z}{Z}_{s}+\omega \mu }.$$

where, *Z*_*s*_ is the equivalent grid impedance of the metasurface. After determining the reflection and transmission coefficients, the z-axial component of electric and magnetic field in a closed form on both sides of the metasurface can be written as:

15 $${H}_{z}=\frac{-{m}_{1}}{4\pi }{\int }_{0}^{\infty }\,d{k}_{\rho }{k}_{\rho }^{2}{H}_{0}^{\mathrm{(1)}}({k}_{\rho }\rho )({e}^{-{k}_{\rho }z}-\frac{\omega \mu }{2{k}_{z}{Z}_{s}+\omega \mu }{e}^{{k}_{\rho }z}){e}^{-{k}_{\rho }{d}_{1}}.$$

for −*d*_1_ < *z* < 0 and

16 $${H}_{z}=\frac{-{m}_{1}}{4\pi }{\int }_{0}^{\infty }\,d{k}_{\rho }{k}_{\rho }^{2}{H}_{0}^{\mathrm{(1)}}(\frac{2{k}_{z}{Z}_{s}}{2{k}_{z}{Z}_{s}+\omega \mu }){e}^{-{k}_{\rho }(z+{d}_{1})}.$$

for *z* > 0.

Note that in the above equations, to simplify further analysis, we assume that the distance between the coils is deeply sub-wavelength. Since we are interested only in the near-field region around it, only Fourier components with $|{k}_{\rho }|\gg {k}_{0}$ are important^13^. Under this assumption, the following approximation can be made:

17 $$-j{k}_{z}=|{k}_{\rho }|$$

We can investigate the electromagnetic interaction between two magnetic dipoles base on the coupled theory^13^ which determines the flux through coil m is expressible through the currents across all of the participating coils.

18 $${{\rm{\Phi }}}_{m}\cong ({\bf{B}}\cdot \widehat{n}){A}_{m}=\sum _{n=1}^{2}\,{L}_{mn}{i}_{n}.$$

where, *A*_*m*_ is the area of the m-th coil, B represents the magnetic flux density in the center of the dipole and *L*_*mn*_ is called self-inductance for *m* = *n* and mutual inductance for *m* ≠ *n*. So, we calculate the flux through coil 2 to obtain the mutual inductance as follows:

19 $${{\rm{\Phi }}}_{2}={B}_{z}(\rho =0,z={d}_{2}){A}_{2}=\frac{-{i}_{1}{\mu }_{0}{A}_{1}{A}_{2}}{4\pi }{\int }_{0}^{\infty }\,d{k}_{\rho }{k}_{\rho }^{2}(\frac{2j{k}_{\rho }{Z}_{s}}{2j{k}_{\rho }{Z}_{s}+\omega \mu }){e}^{-{k}_{\rho }({d}_{1}+{d}_{2})}.$$

In deriving (19), we have used small argument features of Hankel function. The mutual inductance coefficient is obtained from (19) by dividing out the current *i*_1_ as follows:

20 $${L}_{21}=\frac{{{\rm{\Phi }}}_{2}}{{i}_{1}}=\frac{-{\mu }_{0}{A}_{1}{A}_{2}}{4\pi }{\int }_{0}^{\infty }\,d{k}_{\rho }{k}_{\rho }^{2}(\frac{2j{k}_{\rho }{Z}_{s}}{2j{k}_{\rho }{Z}_{s}+\omega \mu }){e}^{-{k}_{\rho }({d}_{1}+{d}_{2})}.$$

Solving the integral of (20), we can obtain an analytical expression for the mutual inductance which depends on the impedance grid (metasurface), after relatively simple calculation of (20), the following is yielded:

21 $${L}_{21}=\frac{-j{\mu }_{0}{A}_{1}{A}_{2}}{32\pi {Z}_{s}^{3}}((\frac{2j{Z}_{s}{\omega }^{2}{\mu }_{0}^{2}}{d}+\frac{4{Z}_{s}^{2}\omega {\mu }_{0}}{{d}^{2}}-\frac{16j{Z}_{s}^{3}}{{d}^{3}})-{(\omega {\mu }_{0})}^{3}{e}^{\frac{d\omega {\mu }_{0}}{2j{Z}_{s}}}{E}_{1}(\frac{d\omega {\mu }_{0}}{2j{Z}_{s}})).$$

where, *d* = *d*_1_ + *d*_2_ is the distance between the coils and *E*_1_(*x*) is the exponential integral.

22 $${E}_{1}(x)={\int }_{x}^{\infty }\,\frac{{e}^{-t}}{t}dt.$$

To highlight the effect of the metasurface on the mutual inductance, one should compare *L*_21_ given by (21) to its value in the absence of the metasurface.

23 $${L}_{21}^{vac}=-\,\frac{{\mu }_{0}{A}_{1}{A}_{2}}{2\pi {d}^{3}}.$$

### The effect of the metasurface on self-inductance

The effect of the metasurface on self-inductance can be investigated in the point dipole approximation via the given approach in^13^. We can determine the metasurface contribution to self-inductance *L*_11_ using the following expression:

24 $${L}_{11}={L}_{11}^{\mathrm{(0)}}-{\mu }_{0}{A}_{1}^{2}{G}_{zz}^{{\rm{ref}}}.$$

where, ${L}_{11}^{\mathrm{(0)}}$ is the self-inductance of coil 1 in free space and ${G}_{zz}^{{\rm{ref}}}$ is the reflected portion of the Green’s function corresponding to the reflected magnetic field due to the metasurface.

25 $${G}_{zz}^{{\rm{ref}}}=\frac{1}{4\pi }{\int }_{0}^{\infty }\,d{k}_{\rho }{k}_{\rho }^{2}(\frac{\omega \mu }{2j{k}_{\rho }{Z}_{s}+\omega \mu }){e}^{-2{k}_{\rho }{d}_{1}}.$$

From the relationship Φ_1_ = *L*_11_*i*_1_ and using (24) and (25), the self-inductance affected by the metasurface ${L}_{11}^{\mathrm{(1)}}$ can be written as:

26 $${L}_{11}^{\mathrm{(1)}}=-\,\frac{j\omega {\mu }_{o}{A}_{1}^{2}}{32\pi {Z}_{s}^{3}}(\frac{j\omega {\mu }_{0}{Z}_{s}}{{d}_{1}}+\frac{{Z}_{s}^{2}}{{d}_{1}^{2}}-{(\omega {\mu }_{0})}^{2}{e}^{\frac{\omega {\mu }_{0}{d}_{1}}{j{Z}_{s}}}{E}_{1}(\frac{\omega {\mu }_{0}{d}_{1}}{j{Z}_{s}}))\mathrm{.}$$

The similar expression for receiving coil ${L}_{22}^{\mathrm{(2)}}$ is obtained by replacing *d*_1_ and *A*_1_ in (26) with *d*_2_ and *A*_2_.

### Wireless power transfer efficiency analysis

In this section, we analytically study the wireless power transfer efficiency improvement in the presence of a metasurface. Figure 2 indicates the equivalent circuit diagram of the magnetic resonant coupling-based wireless power transfer system displayed in Fig. 1. The efficiency of this system can be written as^13^:

27 $$\eta =\frac{{R}_{2}}{{R}_{2}^{{\rm{eff}}}}\frac{\chi }{1+\chi }$$

where,

28 $$\chi =\frac{{R}_{2}^{{\rm{eff}}}{\omega }^{2}|{L}_{21}{|}^{2}}{{R}_{1}^{{\rm{eff}}}|{Z}_{2}{|}^{2}}$$

and

29 $${R}_{1}^{{\rm{eff}}}={\rm{Re}}({Z}_{1}^{{\rm{ef}}f})={R}_{1}-\omega {\rm{Im}}({L}_{11})+{\omega }^{2}{\rm{Im}}(\frac{{L}_{12}{L}_{21}}{{Z}_{2}})$$

30 $${R}_{2}^{{\rm{eff}}}={\rm{Re}}({Z}_{2}^{{\rm{eff}}})={R}_{2}-\omega {\rm{Im}}({L}_{22})+{\omega }^{2}{\rm{Im}}(\frac{{L}_{12}{L}_{21}}{{Z}_{1}})$$

Equation (27) estimates the WPT efficiency close to 100%, when *χ* is as large as possible and the ratio $\frac{{R}_{2}^{{\rm{eff}}}}{{R}_{2}}\ge 1$ as small as possible^13^. At the resonant frequency of the resonators, we obtain (31) by eliminating Im(*Z*_2_) to minimize |*Z*_2_|

31 $$\chi =\frac{{\omega }^{2}|{L}_{21}{|}^{2}}{{R}_{1}^{{\rm{eff}}}{R}_{2}^{{\rm{eff}}}}$$

So we can calculate the power transfer efficiency as follows:

32 $$\eta =\frac{{R}_{2}}{({R}_{2}+\omega {\rm{Im}}(\frac{j\omega {\mu }_{0}{A}_{2}^{2}}{32\pi {Z}_{s}^{3}}(\frac{j\omega {\mu }_{0}{Z}_{s}}{{d}_{2}}+\frac{{Z}_{s}^{2}}{{d}_{2}^{2}}-{(\omega {\mu }_{0})}^{2}{e}^{\frac{\omega {\mu }_{0}{d}_{2}}{j{Z}_{s}}}{E}_{1}(\frac{\omega {\mu }_{0}{d}_{2}}{j{Z}_{s}}))))}\frac{\chi }{1+\chi }$$

where,

33 $$\chi =\frac{{\omega }^{2}{|\frac{-j{\mu }_{0}{A}_{1}{A}_{2}}{32\pi {Z}_{s}^{3}}((\frac{2j{Z}_{s}{\omega }^{2}{\mu }_{0}^{2}}{d}+\frac{4{Z}_{s}^{2}\omega {\mu }_{0}}{{d}^{2}}-\frac{16j{Z}_{s}^{3}}{{d}^{3}})-{(\omega {\mu }_{0})}^{3}{e}^{\frac{d\omega {\mu }_{0}}{2j{Z}_{s}}}{E}_{1}(\frac{d\omega {\mu }_{0}}{2j{Z}_{s}}))|}^{2}}{{f}_{1}\times {f}_{2}}$$

where,

34 $${f}_{1}=(({R}_{1}+\omega {\rm{Im}}(\frac{j\omega {\mu }_{0}{A}_{1}^{2}}{32\pi {Z}_{s}^{3}}(\frac{j\omega {\mu }_{0}{Z}_{s}}{{d}_{1}}+\frac{{Z}_{s}^{2}}{{d}_{1}^{2}}-{(\omega {\mu }_{0})}^{2}{e}^{\frac{\omega {\mu }_{0}{d}_{1}}{j{Z}_{s}}}{E}_{1}(\frac{\omega {\mu }_{0}{d}_{1}}{j{Z}_{s}})))))$$

35 $${f}_{2}=(({R}_{2}+\omega {\rm{Im}}(\frac{j\omega {\mu }_{0}{A}_{2}^{2}}{32\pi {Z}_{s}^{3}}(\frac{j\omega {\mu }_{0}{Z}_{s}}{{d}_{2}}+\frac{{Z}_{s}^{2}}{{d}_{2}^{2}}-{(\omega {\mu }_{0})}^{2}{e}^{\frac{\omega {\mu }_{0}{d}_{2}}{j{Z}_{s}}}{E}_{1}(\frac{\omega {\mu }_{0}{d}_{2}}{j{Z}_{s}})))))$$

**Figure 3:**
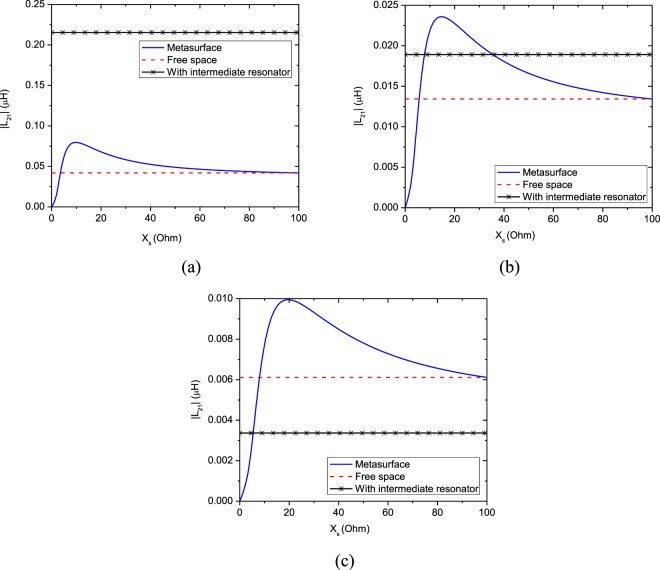
Amplitude of mutual inductance versus equivalent surface inductive reactance at different distance between two dipoles. (**a**) 0.02*λ* (**b**) 0.03*λ* (**c**) 0.04*λ*.

**Figure 4:**
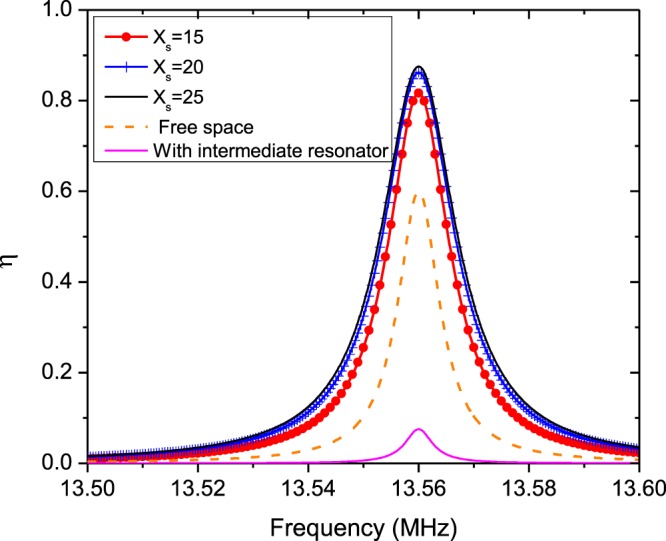
The calculated WPT efficiency between two magnetic dipoles that are 130 cm apart for different equivalent surface impedance.

**Figure 5:**
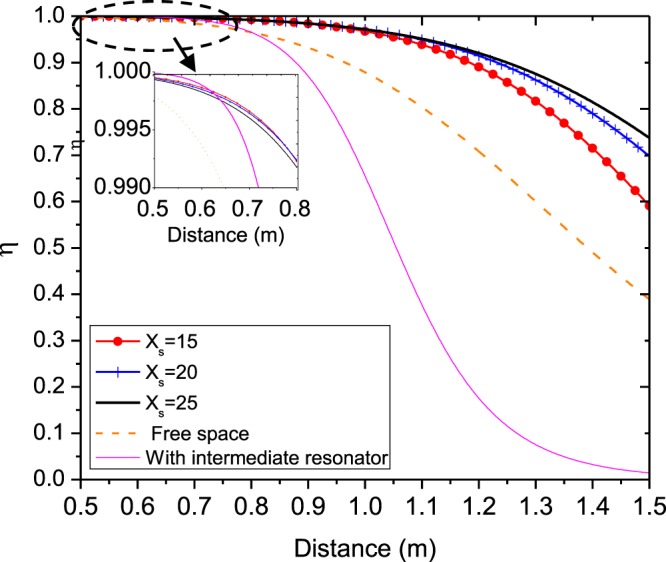
WPT efficiency variation relative to distance between two magnetic dipoles at *f*_0_ = 13.56 MHz.

As an example, consider a setup of the metasurface-enhanced WPT including two coils with radius *λ*/100 separated by distance *d*. The distance of the transmitter and receiver coils from the metasurface is identical and equal to *d*_1_ = *d*_2_ = *d*/2, so the metasurafce is positioned in the middle of the interval between the two coils. For simplicity, the metasurface is assumed to be lossless and less dispersive. Therefore, the equivalent surface impedance of the metasurface is reduced to a purely capacitive or inductive quantity *Z*_*s*_ = $\mp$*jX*_*s*_. Equation (31) confirms that the maximum value of *χ* occurs when the value of |*L*_21_| is maximum. It can be shown that Eq. (31) has a maximum value by choosing a proper surface impedance at a specified distance and resonant frequency. Figure 3 illustrates the variation of |*L*_21_| versus the equivalent inductive reactance of the metasurface for different distances between the two coils. Also, as seen in this figure, we investigated an other condition when a similar intermediate resonator is placed between the center of transmitter resonator and receiver resonator. In this case, assuming there is no coupling between the transmitter dipole and receiver dipole, the equivalent mutual inductance between them due the intermediate dipole can be approximated in the form ${L}_{21}^{\ast }={({L}_{21}^{vac})}^{2}/{L}_{11}$, where ${L}_{21}^{vac}=\frac{(-{\mu }_{0}{A}_{1}{A}_{2})}{(2\pi {(d/\mathrm{2)}}^{3})}$ and *L*_11_ is the self-inductance of transmitter dipole. As shown in Fig. 3 WPT efficiency in this case is bigger than free-space and embedded metasurface cases for distances *d* < 0.03*λ*_0_. But, for *d* > 0.03*λ*_0_, WPT efficiency is maximum for WPT system which a metasurface is embedded between dipoles, because in far distance the metasurface occupies a bigger space and acts as a lens that refocus the evanescent near-field at the center of receiver dipole. As a part of the transmitting energy is suppressed in the intermediate resonator so for *d* > 0.04*λ*_0_ the mutual inductance between the transmitter and receiver dipoles in the case of “with intermediate resonator” is the least value. So the intermediate resonators are usually used for realization of the multi-relay domino WPT system for controlling and guiding the power transfer route^1^. Then, |*L*_21_| and consequently parameter *χ* has a maximum value in the metasurface-enhanced WPT, which is larger than one in the WPT without metasurface. Thus, we expect that the WPT efficiency via (32) in WPT through a metasurface be larger with equivalent surface impedance *Z*_*s*_ = *j*25 Ω compared to WPT without metasurface. Substituting parameter *χ* using (31) for *Z*_*s*_ = *j*25 Ω in (32), we can calculate WPT efficiency as presented in Fig. 4. WPT efficiency versus frequency is represented in Fig. 4. As can be seen from Fig. 4, WPT efficiency is maximum at the resonant frequency of the similar resonator as transmitter and receiver coils. However, this maximum for WPT integrated with a metasurface is larger than one in the absence of metasurface. The calculated WPT efficiency for different equivalent surface impedances at 13.56 MHz is revealed in Fig. 5. As found from Fig. 5, the variation of WPT efficiency versus distance between the two coils for the surface impedance calculated from Fig. 3 has a maximum value, especially at midrange of the distance between the two magnetic dipoles. WPT efficiency for free space (without metasurface) decreases with ratio $\sim 1/{d}^{6}$, while this factor slowly falls in the presence of the metasurface. Thus, we can improve WPT efficiency at different distances using a proper metasurface calculated from our proposed theory as shown in Figs 4 and 5.

### Metasurface Design

**Figure 6:**
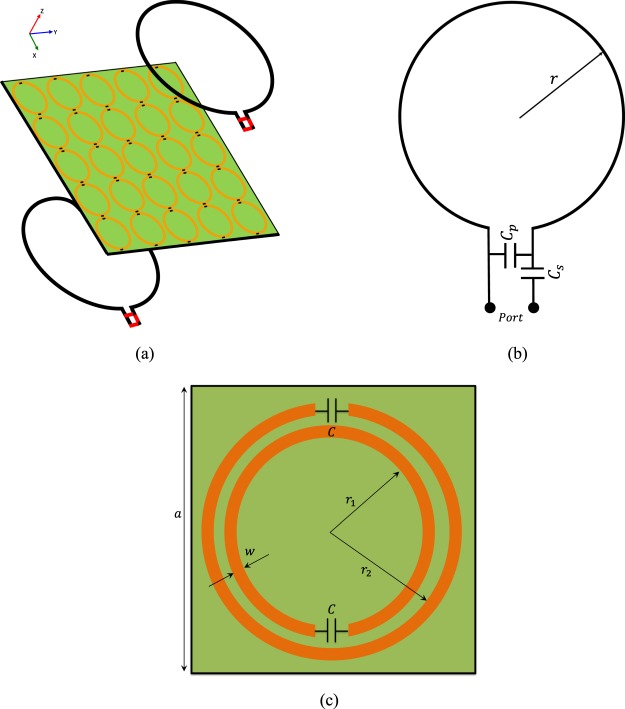
Schematic of WPT system with a metasurface. (**a**) The general structure in HFSS. (**b**) The transmitter and receiver loop antenna geometry. (**c**) Schematic diagram of unit cell of CLSRRs.

To validate our theory, we performed a full-wave simulation for analyzing a WPT system, including two circular loop antennas, as demonstrated in Fig. 6. For practical considerations to match the loop antennas to 50 Ω and also to adjust the resonant frequency, a matching network including two capacitors was used. The capacitance values of *C*_*s*_ and *C*_*p*_ can be calculated through the expressions given in^35^. In this design, the radius of the loop antennas is 20 cm and diameter of the PEC wire is 1 cm. Also, the values of *C*_*s*_ and *C*_*p*_ are calculated as *C*_*s*_ = 30 pF and *C*_*p*_ = 106 pF at the resonant frequency of 13.56 MHz. Next, to investigate the effect of metasurface on the WPT efficiency, we designed a proper metasurafce based on our proposed theory in the previous section. In this section, we designed single-sided CLSRRs^31^ to achieve magnetic lensing and the required equivalent surface impedance. The designed metasurface had both the advantage of compact size and easy fabrication for the inductive WPT system. To design the metasurface, We use HFSS software to design the proposed metasurface. In fact, to design the infinite array of the constitutive unit cells of the metasurface, we calculate Z-parameter using the HFSS to obtain the equivalent surface impedance of the metasurface calculated from our theory (*X*_*s*_ = *j*25 Ω). In our design, we define the geometric dimensions as parametric variables in HFSS then this unit cell is simulated and optimized in a three dimensional environment by assigning Floquet ports on its top and bottom surfaces for a normally incident plane-wave excitation and terminating its sides by periodic boundary conditions to simulate an infinite array. The conductivity of 5.8 × 10^7^ Siemens/m is set for the copper. The final optimized geometry of the constituting sub-wavelength elements of the metasurface are given by *a* = 12 cm, *r*_1_ = 4.86 cm, *r*_2_ = 5.3 cm, *w* = 0.22 cm. The value of the capacitor added to the CLSRRs was *C* = 355 pF. The whole metasurface was etched on a lossy and low profile substrate namely FR-4 with effective permittivity *ε*_*r*_ = 4.4, loss tangent *tanδ* = 0.02 and thickness of 1 mm.

## Discussion

**Figure 7:**
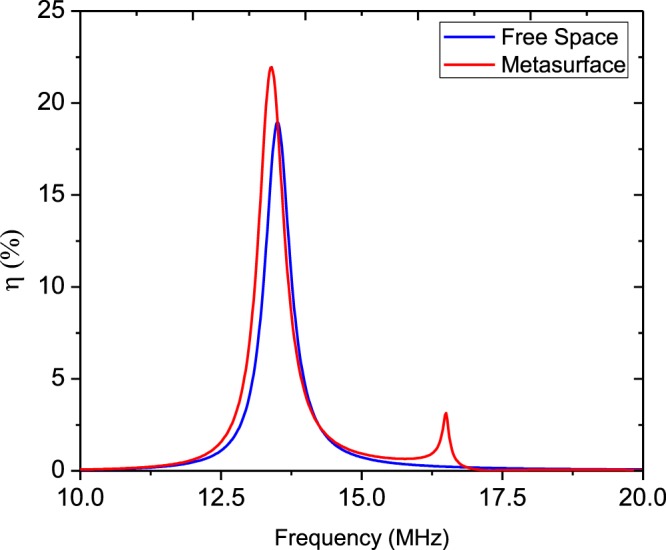
The simulated WPT efficiency between two coils that are 70 cm apart in the presence of the metasurface and in the absence of the metasurface.

We have simulated a WPT system with the same specifications as described above using HFSS, in the presence of the metasurface and without the metasurface, respectively. In our EM simulation, the distance between the transmitter and receiver coils is 70 cm. The WPT efficiency of this system can be calculated via expression |*S*_21_|^2^. The WPT efficiency versus frequency is revealed in Fig. 7 with and without the proposed metasurface, respectively. As predicted and shown in Fig. 7, the maximum of the WPT efficiency occurs at the resonant frequency of the resonators. The results of Fig. 7 indicate a specific clarify a specific feature which is directly related to the effect of the metasurface on WPT efficiency improvement. The energy transfer enhancement for the WPT system has been seen at 13.65 MHz. As shown in Fig. 7, the maximum value of the WPT system has been 18.1% for free-space case (without metasurface), whereas this value has been 22.5% when the proposed metasurface is inserted between the two coils. The metasurface inserted between the two coils was made of an array of 5 × 5 unit cells and positioned in the middle of the transmitter and receiver antennas.

**Figure 8:**
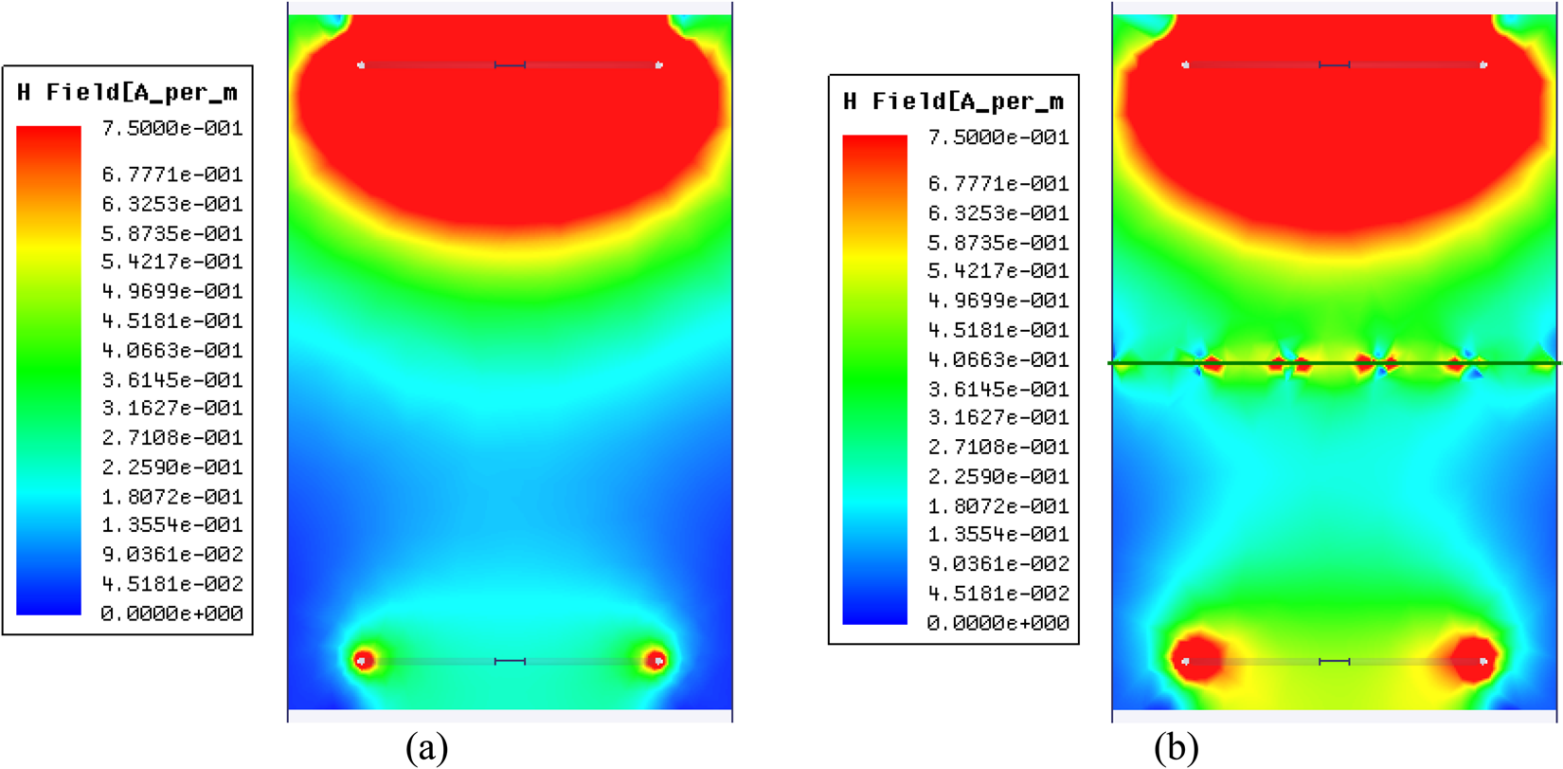
The simulated magnetic field distribution between two coils at *d* = 70 cm. (**a**) Without metasurface. (**b**) With metasurface.

Figure 8 displays the H-field magnitude distribution for the WPT system with and without the metasurface. When the metasurface is inserted between two coils, strong surface waves existing on both sides of the metasurface are observed, which are responsible for the increased magnetic coupling. As presented in Fig. 8 and since the power is transferred between the coils via coupling of evanescent fields, by amplifying the near evanescent field which can be achieved via inserting the proposed metasurface. The electric field and magnetic field can be decoupled in the operating regime of the WPT systems. Since most of the WPT systems are based on magnetic field coupling when an incident electromagnetic wave with a magnetic field is perpendicular to the plane of the metasurface, each unit cell acts as a serial RLC resonator circuit which obeys the frequency dispersive Lorentz model to produce an effective negative permeability^36^.

## Conclusion

We have investigated an analytical theory to improve non-radiative WPT efficiency using metasurfaces instead of metamaterial slabs. In our analysis, we have shown that using a proper equivalent inductive surface impedance embedded between the two magnetic dipoles of the WPT system, a metasurface could be designed for refocusing the flux due to the transmitter coil at the receiver coil and surface wave amplification. Indeed, our analytical expressions have confirmed that WPT efficiency can be improved by designing a proper metasurface, which is predictable. The calculated mutual inductance in the presence of a metasurface is a function of coil size, distance between two coils and equivalent surface impedance. In the previous works, the mutual inductance coupling enhancement has been accomplished by virtue of negative permeability lenses added at the resonant near-filed WPT system. However in this work, we have found that WPT mutual inductance coupling could be improved by inserting a metasurface which has been designed using our proposed theory. In the future works, WPT system performance can be improved by amplifying the near evanescent field which can be achieved via inserting novel meta-lenses at MHz regime.
